# Risk of Gallstone Formation in Aberrant Extrahepatic Biliary Tract Anatomy: A Review of Literature

**DOI:** 10.7759/cureus.10009

**Published:** 2020-08-25

**Authors:** Ademola S Ojo, Alicia Pollard

**Affiliations:** 1 Department of Anatomical Sciences, St. George's University School of Medicine, St. George's, GRD

**Keywords:** gallstone, extrahepatic, biliary tract, anatomical variation

## Abstract

The age-long mnemonic of '5Fs' (fat, female, fertile, forty, and fair) has traditionally been used in medical school instructions to describe the risk factors for gallstone disease. However, evidence suggests that aberrant extrahepatic biliary tract (EHBT) anatomy may contribute significantly to the risk of gallstone disease. This review explores the anatomy and embryological bases of EHBT variations as well as the prevalence of these variations. Also, we discuss the risk factors for gallstone formation in the relationship between gallstone disease and aberrant EHBT anatomy.

## Introduction and background

Gallstone disease is one of the most common abnormalities of the gastrointestinal system with geographical variation in prevalence. About 20 million people (15% of the population) are estimated to have gallstones in the USA [1]. Meanwhile, In Europe, ultrasound studies suggest a prevalence of 9%-21% [2]. The rising incidence of gallstone disease in western countries is considered to be a consequence of increasing obesity rates [3]. The age-long mnemonic of '5Fs' (fat, female, fertile, fair, and forty) has traditionally been used in medical school instructions to describe the risk factors for gallstone disease. However, evidence suggests aberrant extrahepatic biliary tract (EHBT) anatomy may contribute significantly to the risk of gallstone disease [4-11]. The EHBT is made up of the right and left hepatic ducts, common hepatic duct (CHD), common bile duct (CBD), gallbladder, cystic duct, and CBD. Variations in EHBT anatomy have been reported in imaging, cholecystectomy, endoscopic retrograde cholangiopancreatography, as well as autopsy studies which together account for a prevalence of 1.6% to 47.2% [12]. In this review, we discuss the anatomical and embryological bases of EHBT variations as well as explore the various types and prevalence of these variations. Also, we review the risk factors for gallstone formation and the link between gallstone disease and aberrant EHBT anatomy.

## Review

Anatomy and embryology of the EHBT

The liver, gallbladder, and biliary ducts arise as a ventral diverticulum of the endodermal lining of the distal foregut in the 4th week of intrauterine development [13]. This Y-shaped diverticulum grows into the septum transversum, with cellular proliferation at the distal end forming the liver, while the initial diverticulum from the foregut endoderm becomes the bile duct and the distal branches becomes the right and left hepatic duct [14]. A ventral diverticulum from the bile duct proliferates and becomes the gallbladder, with the connecting stalk narrowing to form the cystic duct [13]. Duodenal rotation ensures that the final position of the bile duct is posterior to the duodenum. Initially, the gallbladder and biliary ducts are tubular, however, epithelial lining proliferation results in the formation of a solid core of tissue which recanalizes to establish a definitive lumen [14].

The normal anatomy of the extrahepatic tract can be described as (1) Two hepatic ducts emerging from the porta hepatis; the right and left, uniting to form the CHD. (2) A CHD which receives the cystic duct at an acute angle on its right lateral side midway between the porta hepatis and ampulla of Vater to form the CBD. (3) A single cystic duct which connects the CHD with the gall bladder with an average length between 2-4 cm. (4) A single gallbladder which continues into the cystic duct and (5) a CBD which runs behind the duodenum and pancreas, joining the pancreatic duct and draining into the posteromedial aspect of the second part of the duodenum, measuring 5-20 cm in length [15-17].

Variations in EHBT anatomy

Aberrant embryologic development is responsible for variations in EHBT anatomy. It could manifest as differences in number, length, diameter, or course of any of the components of the EHBT.

Gallbladder

Duplications, septation, agenesis, fundic cap (Phrygian cap), and ectopia are reported variations in gallbladder anatomy [14]. Duplications or triplications of the gallbladder is a rare anomaly, with duplications reported in one in 3800 autopsies [18]. Gallbladder triplications are even less common, which similar to duplications result from subdivision of hepatic diverticulum during embryologic development [19]. Gallbladder septation may occur when longitudinal or transverse thin septa partition the gallbladder into two or more communicating compartments, with an incidence of about 4% [20]. Other abnormalities of the gallbladder include agenesis, with an incidence of 10-65 per 100,000; ectopic gallbladder, found in one in 1600 autopsies; and Phrygian cap found in 4% of the population [18,21,22].

Cystic Duct

Variations in the anatomy of the cystic duct are common findings in imaging studies and cholecystectomy reports, occurring in 18%-23% of individuals [16,23]. Variations in number, length, course, and pattern of entry of the cystic duct into the bile duct have been reported. Absent cystic duct with direct drainage of the gallbladder into the CHD, as well as duplication of the cystic duct, are extremely rare with an unknown incidence [14]. The incidence of a short cystic duct (<5 mm) has been reported as 1.7%-2.6% [16,24]. A long cystic duct is usually associated with abnormalities of course and/or insertion into the CHD. Such a long cystic duct could have a parallel or spiral course about the CHD. Both are associated with variations in insertion into the CHD. Spiral course with medial insertion occurs in 16%-18% of individuals, while anterior or posterior insertion occurs in 22%-32% [16,23]. This insertion could occur close to the porta hepatis (high insertion) or close to the ampulla of Vater (low insertion) with an incidence of 5.5% and 9% respectively [36%]. Anomalous drainage of the cystic duct into the right or left hepatic duct is found in 1%-2% of the population [23]. 

Hepatic Ducts

Though very rare, direct drainage of the right or left hepatic ducts into the gallbladder has been reported [25]. The right hepatic duct could also drain into the neck of the gallbladder or cystic duct, while drainage of the left hepatic duct into the stomach or duodenum has been reported [26,27]. However, these variations are rare. Accessory hepatic ducts are biliary ducts that drain a sector (segment) of the liver, often as sole drainage, into the extrahepatic bile duct, cystic duct, or directly into the gallbladder [28]. Most accessory hepatic ducts arise from the right liver lobe [14]. Autopsy and cholangiography studies reported their incidence as 9%-16% [29,30]. Communicating accessory hepatic ducts connect two aspects of the EHBT, without directly draining any part of the liver [14]. Such anomalous communications have been reported between the right hepatic duct and the cystic duct, CHD, or the gallbladder [30,31]. However, these variations are rare. 

CBD

There are variations involving the number and location of the opening of the CBD into the duodenum. Accessory CBD also called double CBD (DCBD) is one of such anomalies reported in the literature [32]. The incidence of DCBD is unknown, however, it is considered to be a rare anomaly [33]. It results from disturbances in the process of recanalization of the CBD during embryologic development [33]. This accessory duct usually opens separately into the duodenum, stomach, or pancreatic duct [32]. Another reported anomaly of the CBD is an abnormal termination of a single CBD in the stomach, pyloric canal, duodenal bulb, the third or fourth part of the duodenum, with a majority of such ectopic termination occurring in the third and fourth part of the duodenum [34-37]. The reported incidence of ectopic CBD termination is 1 in 1000 in an endoscopic retrograde cholangiopancreatography study [38].

Risk factors for gallstone formation

Three types of gallstones exist in practice: cholesterol, pigment, and mixed stones. Cholesterol stones account for 10% of all gallstones and result when cholesterol concentration exceeds the solubilizing capacity of bile which leads to the precipitation of cholesterol crystals [3]. They are pale yellow, formed only in the gallbladder and have a cholesterol content ranging from 50% to 100% [39]. There is a higher prevalence of cholesterol stones in the United States and Western Europe, compared to developing countries [40]. The risk of developing cholesterol gallstones increases with age, with the middle to older age predominantly affected. In any population, the risk of gallstone formation is higher in females when compared to men. Conditions that increase the net secretion of cholesterol into bile such as obesity, pregnancy, hyperlipidemia, diabetes mellitus, rapid weight loss, and oral contraceptives, as well as estrogen replacement therapy, increases the risk of cholesterol stone formation [39,40]. Genetic polymorphisms in cholecystokinin receptor A (CCK-AR) and cholesterol 7-alpha-hydroxylase (CYP7A1) genes have been linked with an increased risk of cholesterol stone formation [11].

Pigment stones are darker in color and made up of insoluble calcium salts of unconjugated bilirubin, inorganic calcium salts, and cholesterol, with cholesterol constituting less than 20% of the stone [3]. They are more common in non-western populations and result from conditions that lead to an increase in unconjugated bilirubin in bile [40]. Two types of pigment stones exist; black and brown. Black pigment stones are usually formed in the gallbladder in conditions such as chronic hemolytic disorders, bile acid malabsorption, or liver cirrhosis [39]. Brown stones are formed in the large bile ducts due to infection of the biliary ducts leading to increased deconjugation of bilirubin glucuronide. Mixed stones are the most common form of gallstones, accounting for about 80% of cases [3]. The composition is made up of 20%-50% cholesterol [40]. Either alone or in combination with other risk factors, biliary stasis constitutes a risk factor for the formation of all classes of stones.

Gallstone formation in aberrant EHBT anatomy

Few studies have sought to establish a correlation between aberrant EHBT anatomy and the risk of gallstone formation. Variations in the cystic duct have been mentioned as a potential risk factor for gallstone disease. In a retrospective analysis of patients who had endoscopic retrograde cholangiopancreatography (ERCP), Kubota et al. compared 170 patients with gallstone disease (102 cholecystolithiasis and 68 choledocholithiasis) and 140 patients with normal ERCP examination (control group) [4]. A low entry of the cystic duct was observed more frequently in the gallstone disease group compared to the control group (15.7% vs 2.1%; P<0.01). No relationship was found between the type of course of the cystic duct and having gallstone disease. A similar ERCP study comparing patients with gallstone disease and those without it (250 patient in each group) found that patients with gallstones have significantly (p<0.001) longer and narrower cystic ducts (mean length- 48 mm; mean diameter- 4 mm) than those without stones (mean length-28 mm; mean diameter-7 mm) [5]. Also, an intraoperative cholangiography examination involving 468 patients with gallstone disease shows a low entry of cystic duct was present in 39 patients (8.3%) [6]. In that study, the risk of jaundice and pancreatitis due to gallstones was significantly higher in those with a low entry of cystic duct compare to normal entry. While the study focused on the risk of complications from gallstone disease in different EHBT anatomy, it did not establish a relationship between gallstone disease and EHBT anatomy. Another property of the cystic duct considered as a risk factor for gallstone disease is the pattern of entry into the CHD. Caroli-Bosc et al. examine this and found a significantly higher risk of gallstone disease in those with left entry compared to other patterns of entry [7].

The manner of entry of the CBD into the duodenum has equally been a subject of scrutiny in accessing the risk of gallstone disease in aberrant EHBT anatomy. 18 patients out of 16,651 patients who had ERCP for biliary diseases in a study were found to have an ectopic opening of the bile duct into the duodenum, with all 18 occurring at the duodenal bulb [8]. All the patients had a tapered, narrow, and hook-shaped distal CBD and 10 (56%) of the patients had choledocholithiasis. Another abnormality involving the opening of the CBD into the duodenum is the relationship between the CBD and pancreatic duct as these ducts enter the duodenum. The two ducts open into the duodenum via a common channel or separately. Long common channels (>15 mm) are considered abnormal [9]. In a comparative study of patients with common channels (found in 63% of cases) and those with two separate openings, the risk of gallstone disease was higher in those with separate channels [9]. In that study, individuals with longer common channels (>8 mm) were found to be at a higher risk of gallstone disease. This finding is similar to that of other studies that found a higher prevalence of choledocholithiasis in patients with separate channels compared to individuals with common channels [4,9]. Another variation affecting the manner of entry is the uncommon anomaly of a DCBD. The most common form of this variation is those with one duct opening into the duodenum at the major papillae, while the second duct (accessory CBD) opens into the stomach, duodenum or pancreatic duct [41]. In a descriptive study of 47 patients with an accessory CBD, Yamashita et al. found 13 cases (27.7%) have cholelithiasis, with upper gastrointestinal cancer occurring in 12 cases (25.5%) [10]. This accessory duct lacks a sphincter, which allows regurgitation of intestinal content with an attending increased risk of recurrent infection, calculus formation, and malignant transformation [42].

Variations of the EHBT have mostly been described in terms of the number, length, diameter, course, and pattern of entry. These variables create differences in the orientation of various parts of the EHBT with a potential effect on the flow of bile. Sipahi et al. examined the relationship between the sisto-choledochal angle (SCA) (defined as the angle between two crossing lines at the cystic duct-CBD confluence, one along the axis of the cystic duct and the other extending along the CBD axis) and the risk of gallstone disease using magnetic resonance cholangiopancreatography (MRCP) data [11]. The study found a significant association between increasing SCA and the incidence of gallstone disease with a higher risk in those with an SCA > 90 degrees compared to those with less than 90 degrees (P=0.012). The authors postulated that an increase in SCA could result in higher resistance to the flow of bile due to the spiral folds of the cystic duct. The pattern of entry of the cystic duct into the CHD affects the SCA, as a result, individuals with a low entry of the cystic duct may have a wider SCA. This is, however, hypothetical. By comparing 250 patients with gallstone and another 250 without the condition on ERCP, Deenitchin et al. found that patients with gallstones are more likely to have an acute angle between the gallbladder and cystic duct when compared with those without gallstones (mean angle: 84 degrees vs 119 degrees; p<0.001) [5].

In contrast to the findings in the studies above, Khayat et al. analyzed the data of 120 patients who had ERCP and/or MRCP for biliary diseases and found aberrant anatomical variations in 36 (30%) of the cases [43]. The predominant variations found in the study was short cystic duct (n = 24; 20%), left cystic duct insertion (n=6; 5%), cystic duct inserted into the right hepatic duct (RHD) (n=2; 1.7%), and accessory hepatic duct (n=4; 3.33%). The authors find no significant relationship between EHT anatomy and gallstone disease. Two out of the 120 patients had low insertion of the cystic duct into the CHD.

Impaired gallbladder emptying due to increased resistance to bile flow and bile stasis: the possible link

Most studies on factors that influence bile flow have focused on the physiological factors that affect bile flow such as the effects of cholecystokinin and the autonomic nervous system. However, there is limited data on the effect of anatomical factors on the flow of bile in the extrahepatic biliary system. The biliary system has been described as a pump and conduit system with the mechanics of the components as well as the viscosity of bile playing a role in the development of biliary stasis [44]. Bile stasis due to impaired gallbladder emptying has long been recognized as a predisposition for gallstone formation. A computer-assisted cholescintigraphy study comparing gallbladder emptying in normal subjects and individuals with gallstone disease found defective gallbladder emptying more often in the gallstone disease group, irrespective of the age or gender [45].

The cystic duct is not a mere conduit for bile flow to and from the gallbladder, it affects the pressure difference needed to allow the flow of viscous bile out of the gallbladder and less viscous bile into the gallbladder from the CHD [44]. Also, cystic duct geometry, as well as the presence of the valve of Heister, has been shown to affect the resistance to the flow of bile [44]. Previously mentioned studies reported low cystic duct, ectopic CBD opening, double CBD, separate CBD-pancreatic duct opening into the duodenum, and wide SCA as variations associated with an increased risk of gallstone formation. Only one of the studies reported the observation of gallbladder emptying in patients with these variations. Kubota et al. found 66% of patients with low cystic duct who had no gallstone had impaired gallbladder emptying after the procedure [4]. Besides, individuals with abnormal CBD opening into the duodenum such as those with ectopic opening, double CBD, separate CBD-pancreatic duct entry may have associated sphincter of Oddi dysfunction, a factor which is associated with an increased risk of choledocholithiasis [46]. These suggest multiple factors possibly play a role in increasing the risk of gallstone disease in individuals with aberrant EHBT anatomy.

## Conclusions

Anatomical variations abound in the extrahepatic biliary tract with a potential impact on the flow of bile that could predispose to biliary stasis and gallstone formation. Aberrant cystic duct anatomical relationships such as a low cystic duct, left entry of cystic duct into CHD, narrow cystic/gallbladder angle and wide SCA as well CBD anomalies such as ectopic CBD opening, DCBD, separate-pancreatic duct opening into the duodenum, are reported variations associated with an increased risk of gallstone formation. In addition to clarifying their role in gallstone disease, the knowledge of these variations creates a more objective risk assessment for gallstone disease in individuals with findings of aberrant extrahepatic biliary tract anatomy on imaging studies. There is, therefore, a need for further studies to quantitatively evaluate the rate of flow of bile in various parts of extrahepatic biliary tract anatomy so as to better understand the potential impact of this on gallstone formation.
